# Chronic environmental exposure to lead affects semen quality in a Mexican men population

**Published:** 2013-04

**Authors:** Javier Morán-Martínez, Pilar Carranza-Rosales, Mario Morales-Vallarta, José A. Heredia-Rojas, Susana Bassol-Mayagoitia, Nadia Denys Betancourt-Martínez, Ricardo M. Cerda-Flores

**Affiliations:** 1 *Departamento de Biología Celular y Ultraestructura, Centro de Investigación Biomédica Facultad de Medicina, Universidad Autónoma de Coahuila, Coahuila, México. *; 2 *Facultad de Ciencias Biológicas, Universidad Autónoma de Nuevo León. *; 3 *Departamento de Biología Celular y Molecular, Centro de Investigación Biomédica del Noreste, Instituto Mexicano del Seguro Social, Monterrey, México.*; 4 *Departamento de Biología de la Reproducción, Centro de Investigación Biomédica Facultad de Medicina, Universidad Autónoma de Coahuila, Coahuila, México.*; 5 *Unidad de Biología Molecular, Genómica y Secuenciación. Centro de Investigación y Desarrollo en Ciencias de la Salud, Universidad Autónoma de Nuevo León, Monterrey, México.*; 6 *Facultad de Enfermería, Universidad Autónoma de Nuevo León, Monterrey, México.*

**Keywords:** *Lead*, *Spermatic quality*, *Infertility*, *Environmental*

## Abstract

**Background: **Male infertility is affected by several factors. Lead is one of the heavy metals more bioavailable than usually modifies the sperm quality in humans.

**Objective:** The aim of this study was to establish the role of lead in semen quality in environmentally exposed men.

**Materials and Methods: **Semen and blood samples were obtained from two groups: the exposed group (EG=20) and the non-exposed group (NEG=27). Two semen aliquots were used, one to evaluate spermatic quality and the other for lead determination. Blood (PbB) and semen lead (PbS) determination was performed by atomic absorption spectrophotometry.

**Results:** The PbB concentration was significantly greater in the EG, 10.10±0.97 µgdL-1 than in the NEG, 6.42±0.38 µgdL-1 (p<0.01), as well as the PbS concentration, with 3.28±0.35 and 1.76±0.14µgdL-1 in the EG and NEG respectively (p=0.043). A significant correlation between PbS and PbB concentration in the EG was found (r=0.573, p=0.038). Overall, the spermatic quality was lower in the EG than in the NEG. Specifically, there were significant differences in the spermatic concentration [EG=43.98±6.26 and NEG=68.78±8.51X10^6^ cellmL-1 (p<0.01)], motility [EG=49±7 and NEG=67±4% (p=0.029)], viability [EG=36.32±3.59 and NEG=72.12±1.91% (p<0.01)] and abnormal morphology [EG=67±18 and NEG=32±12% (p<0.01)]. In the immature germ cells (IGC) concentration differences were found only for A cells (EG=8.1±1.1x100 and NEG=3.2±1.9X100 spermatozoa) (p<0.01) and for Sab cells (EG=3.4±2.2x100 and NEG=1.1±1.0X100 spermatozoa) (p=0.041).

**Conclusion:** These results suggest that chronic environmental exposure to low levels of lead adversely affect the spermatic quality.

## Introduction

The infertility problems associated with lead in men environmentally and occupationally exposed have been reported (1-5). The findings of these prior studies are interesting but their results show variability in the association between lead and semen characteristics.

Working in smelters is an important environmental exposure factor for lead. Some authors have found that low or moderate seminal lead levels in smelter workers were correlated with a decrease in the semen quality; also, oligospermia, asthenospermia and an increased frequency of teratospermia have been reported in workers exposed to lead (2, 3). Some smelter workers were found to have hypogonadism and low serum testosterone concentration (1). On the other hand, men occupationally exposed to inorganic lead showed different endocrine and reproductive dysfunctions, particularly at the hypothalamic-pituitary-testicular axis (6-8). In this context, there are not published studies for Mexico that refer to the effects of lead on semen quality.

In order to establish the role of environmental lead exposure on semen quality (sperm quality is defined by the assessment of morphology, motility, viability and number of spermatozoa), we conducted this study in men living within a 500 meter radius of the third largest smelter and lead producer in the world, located in Torreón, State of Coahuila, México.

## Materials and methods

This cross-sectional study was conducted in the Cellular Biology and Ultrastructure Department, Biomedical Research Center, School of Medicine of the Autonomous of Coahuila University. A total of 47 subjects were admitted for clinical and laboratory examinations. They were informed of the study objectives and given informed consent letters to sign (the protocol was approved by the Biomedical Research Center Committee, School of Medicine, University of Coahuila).

Inclusion criteria were age (18-30 years old), healthy, residents of the study area, similar social and economic status, 3 days of sexual abstinence. Exclusion criteria were subjects currently on any medication were not included. Also, patients with varicocele, leucospermia, smokers and alcoholic men were excluded from the study. The subjects of the study had lived in the same area for at least two years. 

Twenty subjects who were residents within 500 meter radius from the metallurgic zone formed the exposed group (EG: n=20), and 27 subjects residing in two communities at a distance of 15 and 18 km, respectively from this zone formed the non-exposed group (NEG). In order to control for possible confounding sources of lead, all subjects were interviewed using a questionnaire, which included items on living conditions, socioeconomic status and occupational factors.

**Sample collection and lead assay**

The semen samples were collected by masturbation after at least 4 days of abstinence. The materials used for sample collection were plastic containers (125 ml), Falcon tubes (conical polystyrene, 15 ml) and polyethyelene tubes (5 ml) (Becton Dikinson Labware, USA). These were cleaned with 10% nitric acid and rinsed with deionized water. The semen specimens were placed in an incubator at 37^o^C immediately after collection. 

After liquefaction, ejaculates were divided into two aliquots. One aliquot was used to carry out routine laboratory analysis: seminal volume, sperm count, total amount of sperm per ejaculate, sperm motility and viability in accordance with the guidelines recommended by the World Health Organization. Smears for IGC and spermatic morphology determinations were stained with the modified Bryan/Leishman stain (9). The second semen aliquot was used for lead determination. Lead analysis in blood and semen samples were measured by atomic absorption spectrophotometry with a heated graphite furnace according to Brown *et al* (10).

**Statistical analysis**

The seminal sperm concentration, progressive sperm motility, percentage of viable sperm and sperm morphology were analyzed by analysis of variance (ANOVA). Immature germ cells (IGC) are considered all round cells not identified as leukocytes, these cells are: spermatogonia, primary and secondary spermatocites and Sab, Scd spermatids. The germinal cell classifications are based on those proposed by Macleod with modifications by WHO (9). The IGC concentration per field was calculated as follows (11):

Concentration ion=Number of IGC SpermCountmL/(100 Sperrmatozoa)

 Plasmatic and seminal lead concentrations were analyzed by ANOVA between groups and by simple correlation. Regression analysis was used to assess the association between lead semen concentration and spermatic quality in the EG. The results are presented as the mean with standard error of mean (SEM). The STATA version 6 program was used for all statistical analysis (12).

## Results

The average age in the EG was 25±1.30 years (range 22-27) and the NEG was 25±1.24 years (range 20-26). Semen lead levels in all subjects were always less than this level in blood and were significantly higher in EG (3.28±0.35 nmol/L) in comparison to NEG (1.76±0.14 nmol/L), p<0.043 (Table I). 

The mean blood lead concentration in the EG was 10.10±0.97 nmol/L and for NEG 6.42±0.38 nmol/L (p<0.01). A significant correlation between the levels of lead in semen and in blood of the EG were observed (r=0.573, p=0.038). No significant correlations (p=0.071) were found between lead semen concentration and seminal parameters (vs. spermatic concentration (X10^6 ^cellmL-1): r=-0.006; vs. spermatic viability: r=0.244 and vs. spermatic motility: r=-0.05). Semen quality parameters in men was affected in all parameters for both groups (Table II). 

A significant higher percentage (p=0.029) of mobile spermatozoa were found in NEG (67±4%) as compared to EG (49±7%). The mean sperm concentration was also significantly higher (p<0.01) in NEG than EG, 68.78±8.51 and 43.98±6.26×10^6 ^cell/mL, respectively. The volume was similar in the NEG (2.9±0.26 mL), compared to EG (3.2±0.32 mL). On the other hand, an increased percentage of morphologic abnormalities were observed in EG (67±18%) compared to 32±12% in NEG (p<0.01) (Table III). 

**Figure 1:**
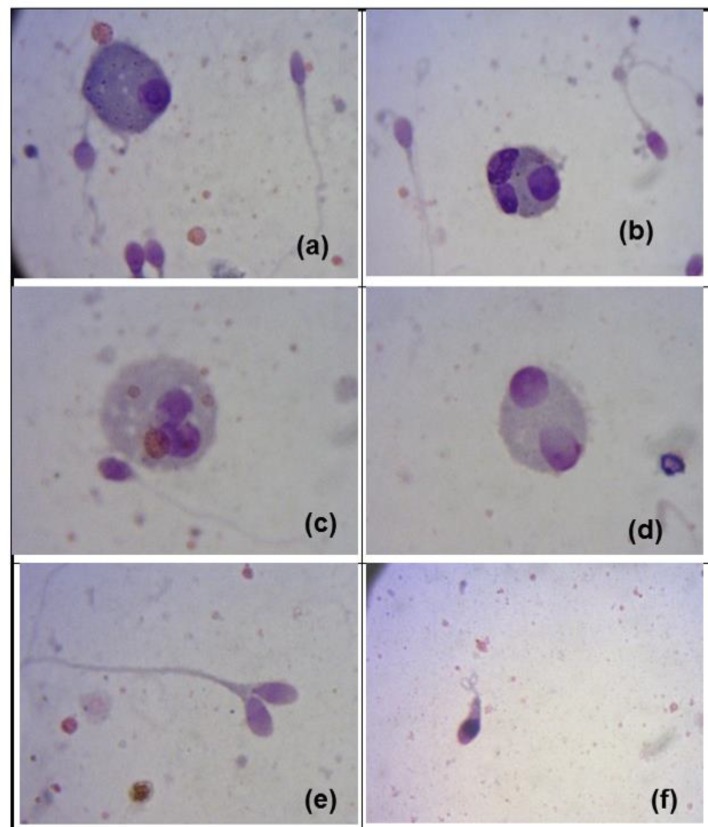
Immature germ cells and sperm morphology in the exposed group.

The predominant abnormalities were defects of the middle piece, including cytoplasmatic droplets (41%), and defects of the head (large oval head (15%), pyriform head (3%) and duplicate head (5%) (Figure 1). Significant differences were found between the percentage of germinal cells secretion observed in the EG in comparison with NEG (global mean by germinal cell by group: p<0.01). The principal germinal cells secretion were spermatogonia type A, and germinal cells as Sab and Scd (Table IV, Figure 1).

**Table I:** Blood and semen lead concentration in the two study groups (Mean±SEM)

**Sample**	**Lead concentration (nmol/L)**			
	**EG**	**Range**	**NE** **G**	**Range**
Blood	10.10 ± 0.97**	(4.4-22.7)	6.42 ± 0.38	(4.9-13.7)
Semen	3.28 ± 0.35*	(0.86-7.44)	1.76 ± 0.14	(0.66-2.60)

**p<0.01, *p<0.05 EG: exposed group, NEG: non-exposed group

**Table II:** Seminal quality in the study groups (Mean±SEM

**Seminal parameters**	**NEG**	**EG**
Concentration (X10^6^cells/mL)	68.78 ± 8.51**	43.98 ± 6.26
Motility (%) a	67 ± 4*	49 ± 7
Viabiltiy (%) b	72.12 ± 1.9*	36.32 ± 3.59
Morphology (%) c	33 ± 12	68 ± 18**
Volume (ml)	2.9 ± 0.26	3.2 ± 0.32

a) Show total motility percent by group. b) Show the live motile and live immotile spermatozoa percent by group. c) Show normal morphology percent by groups. *p<0.05, **p<0.01 EG: exposed group, NEG: non-exposed group

**Table III:** Spermatic morphology determination in the study groups (Mean±SEM

**Form**	**NEG (%)**	**EG (%)**
Normal	68**	33
Large oval head	11	18*
Small oval head	8**	1
Amorphous middle piece	11	41**
Duplicate head	1	5*
Duplicate tail	1	2

*p<0.05; **p<0.01 EG: exposed group, NEG: non-exposed group

**Table IV:** Principal immature germ cells concentration in the study groups (Mean±SEM

**Cell Type**	**NEG (X100 spermatozoa)**	**EG (X100 spermatozoa)**
Spermatogonia A	3.2 ± 2	8.1 ± 1 **
Spermatid Sab	1.1 ± 1	3.4 ± 2 *
Spermatid Scd	2.1 ± 1	2.8 ± 2

*p<0.05, ** p <0.01 EG: exposed group, NEG: non-exposed group

a) Spermatogonium.                    b, c, d) Spermatids Sab.

e) Doublé head                            f) Amorphous head and coiled tail.

## Discussion

The results of our study show that exposure to lead is related to decreased of the quality characteristics in semen in environmental exposed men. In this study, the EG lead blood concentration was higher than NEG. Blood studies made in the same smelter exposed area, show the results in the adult exposed group and control group were 19.2±4.5 and 8.9±1.3nmol/L (p<0.001) respectively, using the same method for lead determination used in this study. On the other hand, in children (7-12 years old) exposed environmentally to lead in the same area a significant difference was found when compared with a control group (17.3±56 and 6.4±3.1 nmol/L respectively) (p<0.01) (13, 14). Garcia-Vargas *et al* showed results in children living nearest school refinery with 27.6 nmol/L mean of lead blood (15). 

These studies show similar results in lead blood and suggest a potential risk of exposition of people, mainly of young and mature men, and its repercussion in health, showing the latent environmental chronic exposure in this population (13-15). On the other hand, our results show a decrease in the seminal parameters in the exposed men for spermatic concentration, asthenospermia and teratospermia. The semen lead concentration in the EG and NEG was 3.28±0.35 nmol/L and 1.76±0.14 nmol/L respectively, these data are similar to those described by Saaranen *et al* which measured semen lead from infertile (3.2 nmol/L) and fertile men (1.7 nmol/L) (16). 

Earlier, Jockenhövel *et al* reported significant results in semen lead concentration in infertile men of 11.18 nmol/L vs. 5.6 nmol/L in fertile men (6). Other results in environmental exposure show 3.8 and 11.10 nmol/L of lead concentration in normal men (17, 18). These similar levels refer that low levels of lead in semen affect seminal characteristics. Butrimovitz *et al* suggested that lead in semen is a more accurate index of the degree of reproductive exposure than lead in blood (17). Our results, are similar to those published by Wyrobeck *et al,* Robins *et al*, Lerda, and Chia *et al*, which found that teratospermia and asthenospermia are principally affected, suggesting that the sperm morphology is the most sensitive parameter to use when studying changes caused by toxic substances during spermatogenesis (3, 19-21). 

The teratospermic index is a good indicator for the status of the testicular germinal epithelium (22). This could be explained because lead interacts with the germinal cell in the seminiferous epithelium, where structural aspects in the spermatozoa development could be altered. Subcellular damage by lead can possibly be caused by the competition that Pb (2^+^) has by replacing Zn (2^+^) in Human Protamine 2 (HP2) *in vivo* and *in vitro,* causing alterations in sperm chromatin condensation and reducing fertility (23, 24). 

Other studies support these results by showing that the take-up of Pb (2^+^) by spermatozoans during testicular development and epididymal transport can alter chromatin condensation (25). Benoff *et al* confirm that metal ions, like Pb (2^+^), compete with Zn (2^+^) inducing alterations in human sperm mannose receptor expression (26). Comparison of sperm count, percentage of abnormal forms, viability and motility demonstrated statistically significant differences based on fertility status. In fact, there are some reports showing interesting and controversial results about morphology damage and semen lead concentration, for example, Jockenhövel *et al*, did not find significant correlation between lead concentration and morphology, however, an increase of lead concentration and amorphous cells were determined (<50% and >50% with 10.87±12.12 and 11.58±1.71 nmol/L; morphology and lead concentration respectively) by Butrimovitz *et al* another study found 42.14±4.1 percent of the abnormal morphology in infertile environmentally exposed men (6, 17, 18). 

In our work, 67% of total cells were abnormal, similar results (57.9%) have been reported by Saaranen *et al* (16). In a literature review, there were no previous reports about the relationship between semen lead concentration and deficient spermatic morphology. The principal germinal cells secretion is shown in the table 4, in our study, significant differences were found between groups, the secretion of type A germinal cells was higher in the EG than in the NEG (8.1±1.12 and 3.2±1.9 respectively) (p<0.01). Similarity, from Sab type cell, the average values were 3.4 ±2.2 in the EG and 1.1±1.0 in the NEG (p=0.041). In literature, there are no reports showing lead and germinal immature cells association. 

A study in normal, oligozoospermic and azoospermic men in this geographical area reports 10.74±11.9; 8.05±16.8 and 19.90±25.4 immature germinal cell concentration respectively (27). These results show a relationship between oligospermic men and the germinal cell concentration, similar data to the ones found in our study, although, the spermatic concentration found in our EG was actually over an oligospermic value (43.98±6.26×10^6^ cellmL-1). 

Lead is a broad-spectrum agent which may exert pronounced effects on different subcellular systems. It is known that Pb influences biological enzyme systems and it can be assumed that numerous mechanisms of interaction are yet to be elucidated. Furthermore, the regulatory mechanisms of the male reproductive system are very complex and also not completely understood. It is likely that Pb interacts with one or more of those mechanisms: it could be on reproductive organs at different levels, or on endocrine control (possible suppression of FSH and LH) of reproduction, or both (28). 

Some actions of lead on the male reproductive system of different species includes infertility, germinal epithelial damage, oligospermia and testicular degeneration, decreased sperm motility, prostate hyperplasia, abnormal sperms, teratospermia, hypospermia and asthenospermia (29). As other heavy metals, lead seems to exert its toxicity upon different subpopulations of testicular cells. Mechanisms of heavy metal toxicity vary and not only include different cell sensitivities, but also direct vs. indirect actions. 

Furthermore, it appears that primary damage to one cell type may secondarily affect other cell types in the testes. For example, in the case of lead, there are direct evidences for testicular toxicity in germinal cells and Leydig cells, but, for Sertoli cells toxicity is possible a secondary effect. The cellular motility in the present study was different for the NEG and the EG (67±4 and 49±7% respectively) (p=0.029), similar results were found in infertile men for this parameter by El-Zohairay *et al* (33.00±5.8%) and Saaranen *et al* (43.9±18.9%) (15, 18).

However, in our work, a positive correlation was not found between lead in semen concentration and seminal parameters. Similar results were found by Saaranen *et al*, Jockenhövel *et al* and Noack-Füller *et al* (6, 16, 30). The smelter has been contaminating this area for more than a hundred years (starting in 1887), it is the first silver producer in the world (year 2003: 78, 575, 158 Oz), and the first zinc (year 2003: 209, 403 tons), lead (year 2003: 125, 735 tons), and gold (year 2003: 1, 056, 385 Oz) producer in Latin America. 

In the studied area, the bioavailability (air) and accumulative availability (dust and soil) of lead concentrations are critical. For example, the lead concentration in total air suspended particles (TSP) and suspended particles of size <10µm (PM-10) were 6.1 and 3.8 µg/m^3^ in a schools near the smelter zone in the same area of our study (15). These values are higher than the parameters suggested by the U.S. National Ambient Air Quality Standard, air values used in Mexico are 1.5 µg Pb/m^3^ (31). In soil and dust, the main problem was in the superficial layers (same on schools), the results show in soil a concentration of 2772.8 µg Pb/g (layer 0-10 cm), 2374.7µg Pb/g (layer 10-20 cm) and dust 3471.3µg Pb/g, these values surpassed the U.S. Environmental Protection Agency parameters (200-500 µg/g soil) (16, 32). 

The environmental exposure in this smelter complex shows a potential and different contamination manner in this population. Allowed concentrations of toxic contaminants are still a debatable issue in developing countries, where there is wide variation in human susceptibility and tolerance, depending on general human health and other environmental factors. Therefore, this chronic exposure on this population in this risk area is critical and there should be other control and prevention strategies. 

## Conclusion

In conclusion, this study shows that the environmental exposure to low levels of lead affect the spermatic quality in an impairing way. Other studies about occupational and environmental lead exposure effects and spermatic quality in this risk area would be very interesting.
